# The Predictive Significance of Platelet‐to‐Lymphocyte Ratio for Miscarriage: A Systematic Review and Meta‐Analysis

**DOI:** 10.1002/iid3.70119

**Published:** 2025-01-07

**Authors:** Xiaoyi Wang, Yanfang Zhao, Yangyang Fan, Yun Liu

**Affiliations:** ^1^ Obstetrics Department Shaanxi Provincial People's Hospital Xi'an Shaanxi China; ^2^ Obstetrics Department Weinan Maternal and Child Health Hospital Weinan Shaanxi China

**Keywords:** early miscarriage, miscarriage, missed miscarriage, platelet‐to‐lymphocyte ratio, prediction

## Abstract

**Background:**

Miscarriage is a common complication of pregnancy, and its underlying pathophysiologic mechanisms remains unclear. The platelet‐to‐lymphocyte ratio (PLR), a prothrombotic and inflammatory marker, has been controversially discussed as a potential predictor of miscarriage. This systematic review and meta‐analysis aimed to assess the predictive significance of the PLR in women with miscarriage compared to healthy pregnancies.

**Material and Methods:**

Relevant studies were systematically searched in PubMed, Embase, Web of Sciencey, and Cochrane Library up to December 31, 2023. A systematic review and meta‐analysis following PRISMA guidelines was conducted. Articles were identified, screened, and evaluated for quality to determine the predictive value of PLR for miscarriage.

**Results:**

Fourteen eligible articles, comprising a total of 3745 patients, were included in the meta‐analysis. The pooled analysis found comparable PLR levels between miscarriage and non‐miscarriage groups (SMD = 0.25; 95% Confidence Interval (CI): −0.05 to 0.54). Subgroup analysis revealed significant differences in PLR levels in the missed miscarriage group (SMD = 0.29; 95% CI: 0.01–0.56). and in studies with sample sizes smaller than 200 (SMD = 0.31; 95% CI: 0.05–0.56). Other subgroups did not exhibit significant differences. Subgroup analysis of PLR levels and miscarriage risk demonstrated no significant differences across all subgroups.

**Conclusion:**

PLR is not a reliable predictor of miscarriage in general. However, for missed miscarriage cases, elevated PLR levels may serve as a practical and cost‐effective marker for prediction.

## Graphical abstract

To our knowledge, this is the first systematic review and meta‐analysis to examine the predictive value of PLR values for miscarriage. There are different pathophysiologic mechanisms in different types of miscarriage, so we conducted subgroup analyses for different types of miscarriage. The subgroup analysis indicated that differences in PLR levels between miscarriage and nonmiscarriage were significant in the missed miscarriage group.

**Abbreviations**
AUC: area under the curve
BMI: body mass index
CBC: complete blood count
NOS: Newcastle–Ottawa Scale
PLR: platelet‐to‐lymphocyte ratio
ROC: receiver operating characteristic
SMD: standardized mean differences

## 1 Introduction

Miscarriage is a significant global concern during pregnancy, with approximately 23 million cases occurring worldwide each year, equating to an average of 44 losses per minute [1]. Miscarriages are broadly classified based on gestational timing: early miscarriages occur before 12 weeks, while late losses occur between 12 and 22 weeks of gestation [2, 3]. Early miscarriage represents the predominant form, accounting for 80% of cases, with the incidence notably decreasing beyond the initial trimester [4]. Miscarriage is not only emotionally distressing for affected women, but it may also result in maternal complications, such as anemia, uterine and endometrial injuries, and infections [5].

Despite extensive research into its underlying causes, the pathophysiological mechanisms driving miscarriage remain poorly understood. Emerging evidence indicates that many miscarriages may be linked to immunological and endocrinological factors, operating through complex interactions within the immune and haematological systems [6, 7]. This has prompted growing interest in the predictive role of complete blood count (CBC) parameters in miscarriage, due to the test's simplicity, affordability, and accessibility. The platelet‐to‐lymphocyte ratio (PLR), calculated as the ratio of platelet count to lymphocyte count, serves as a marker of prothrombotic and inflammatory processes [8].

Elevated PLR has been associated with thrombotic and inflammatory processes [9] and investigated in pregnancy‐related conditions, including gestational diabetes, pancreatitis, pre‐eclampsia, and early premature rupture of membranes [10, 11, 12]. Tola and colleagues hypothesized that the thrombosis of spiral arterioles might underlie the observed inverse relationship between PLR and successful embryonic implantation [13].

A recent meta‐analysis by Hantoushzadeh and colleagues evaluated PLR in women with a history of miscarriage (both missed and threatened) and recurrent pregnancy loss compared to healthy pregnancies. Their analysis of 14 studies found no statistically significant difference in PLR between the pregnancy loss and control groups. While this meta‐analysis provided valuable insights into recurrent miscarriage, it did not address other types of miscarriages and primarily included studies published before 2023. The present study extends this work by providing an updated literature review and conducting comprehensive subgroup analyses based on miscarriage timing and classification [14].

Multiple studies have investigated the predictive value of PLR in pregnancy loss, yielding conflicting results regarding its association with miscarriage risk. Yavuz and colleagues analyzed data from 100 pregnant women and found that although PLR values were higher in cases of missed miscarriage, the difference did not reach statistical significance [15]. In contrast, a study of 285 women (137 with early pregnancy loss and 148 with healthy pregnancies) demonstrated significantly elevated PLR values in the early pregnancy loss group compared to controls, suggesting PLR's potential utility a predictor of early pregnancy loss [16]. Additionally, Yakistiran and colleagues proposed that reduced PLR levels could serve as a practical and cost‐effective marker for predicting miscarriage [17]. Given these contradictory findings, the present meta‐analysis was designed to consolidate and analyze the existing evidence on the predictive capacity of PLR for miscarriage.

## 2 Materials and Methods

This systematic review was carried out following the Preferred Reporting Items for Systematic Review and Meta‐analysis (PRISMA) statement [18], and was registered in advance with the PROSPERO (CRD42024558500).

### 2.1 Search Strategy

A comprehensive literature search was performed in December 2023 across Pubmed, Embase, Cochrane Library, and Web of Science without applying any filters. The search terms were as follows: (“Blood Platelets” OR “Blood Platelet” OR “Platelet, Blood” OR “Platelets, Blood” OR “Thrombocytes” OR “Thrombocyte” OR “Platelets” OR “Platelet”) AND (“Lymphocytes” OR “Lymphocyte” OR “Lymphoid Cells” OR “Cell, Lymphoid” OR “Cells, Lymphoid” OR “Lymphoid Cell”) AND “Ratio” AND (“Miscarrage” OR “Abortion”). The detailed search formula for all databases is provided in File S1. In addition to the database search, the reference lists of selected articles were manually screened to identify further relevant studies.

### 2.2 Criteria for Inclusion and Exclusion

The following inclusion criteria were applied [1]: Observational study design (case–control, cohort, or cross‐sectional) [2]; Study population comprising naturally conceived singleton pregnancies, with clearly defined miscarriage and healthy control groups [3]; Measurement of PLR levels [4]; Absence of pregnancy complications [5]; Publications in English. Exclusion criteria included [1]: Reviews, conference abstracts, and letters [2]; Recurrent miscarriage [3]; Duplicate data [4]; Unavailable full text [5]; Non‐English publications. Two investigators independently reviewed titles and abstracts. Final decisions were made following a full‐text review. Any disagreements between the two investigators were resolved through consultation with a third researcher.

### 2.3 Data Extraction

Primary data extraction from the included studies was undertaken by two researchers. Extracted data encompassed the first author's surname, year of publication, country, study design, matching details for miscarriage types, age, gestational week, and body mass index (BMI). Discrepancies were addressed through deliberation and, if necessary, consultation with a third researcher.

### 2.4 Evaluation of Quality

The quality assessment of the included studies was conducted independently by two investigators using the Newcastle–Ottawa Scale (NOS) [19], focusing on the aspects of Selection, Comparability, and Exposure. A maximum of one star could be assigned in the Selection and Exposure categories, while up to two stars were possible in the Comparability category. The studies were rated on a scale of 0–9, with scores of 0–2 indicating poor quality, 3–5 indicating moderate quality, and 6–9 indicating high quality. Any discrepancies in quality assessments were resolved through discussion.

### 2.5 Statistical Analysis

The meta‐analysis was undertaken using RevMan 5.4.1 software (version 5.4.1; Cochrane Collaboration). Continuous variables were aggregated as standardized mean differences (SMD). Heterogeneity was assessed using forest plots and the *χ* ^2^ and *I* ^*2*^ tests. A random‐effects model was employed for data analysis when the *χ* ^2^ *p* < 0.05 or *I* ^*2*^ was > 50%, indicating significant heterogeneity. Otherwise, a fixed‐effect model was applied. Subgroup analyses based on different countries, sample sizes, gestational age, and types of miscarriage were performed. Additionally, one‐way sensitivity analyses were conducted to assess the impact of individual studies on the overall results for outcomes with significant heterogeneity. Publication bias was assessed both visually and statistically. Funnel plots were generated using RevMan 5.4.1 software, while Egger's regression tests [20] were conducted with Stata version 15.1 (Stata Corp, College Station, TX, USA). A *p* < 0.05 is indicative of statistical significance.

## 3 Results

### 3.1 Selection of Studies

**Figure 1:**
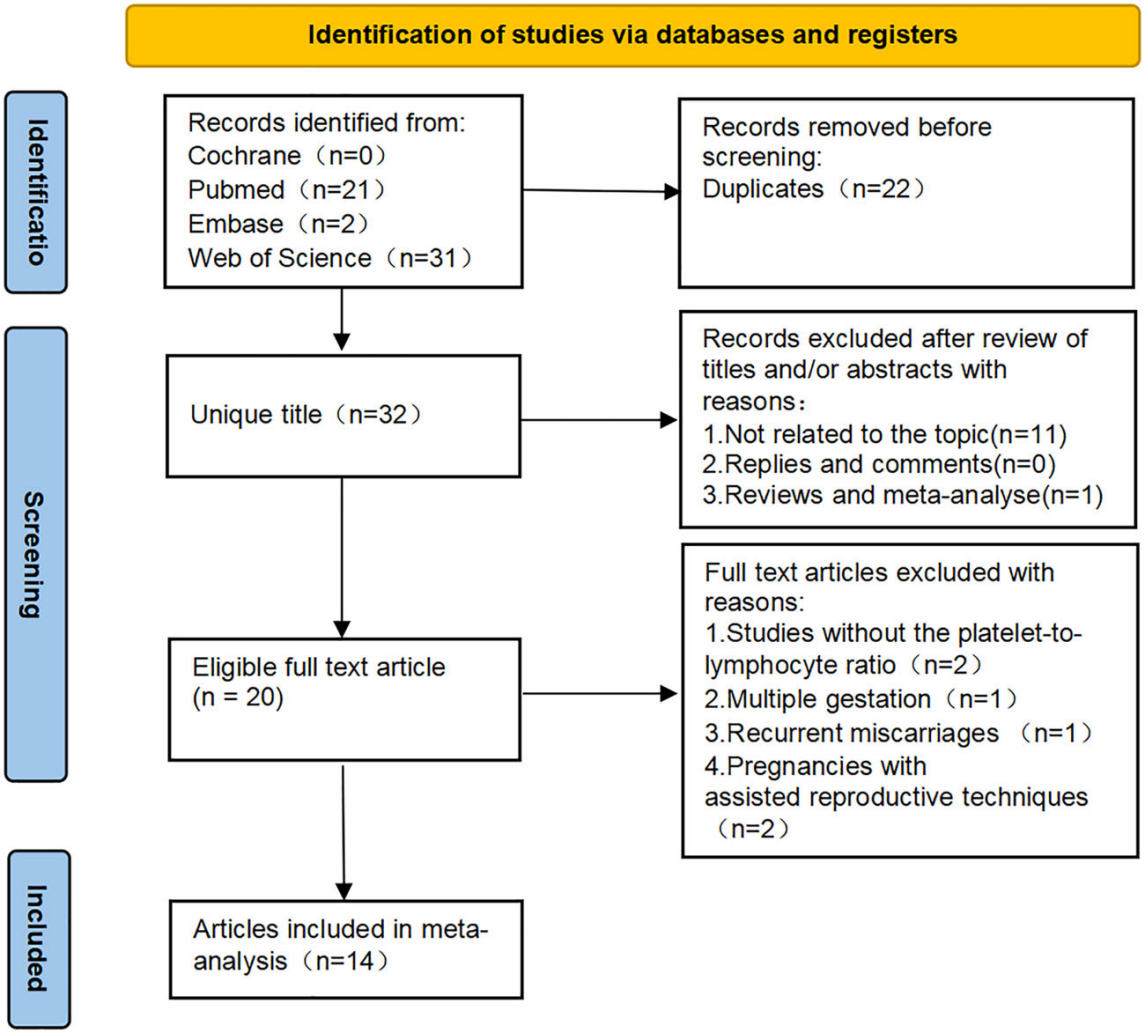
Flowchart of the systematic search and selection process.

The literature search initially identified 54 articles, from which 22 duplicates were removed. The remaining 32 articles were screened based on titles and abstracts, resulting in 20 articles selected for full‐text evaluation. Following a thorough assessment based on the inclusion and exclusion criteria, 14 full‐text articles, encompassing 3745 patients, were included in the meta‐analysis [15, 16, 17, 21, 22, 23, 24, 25, 26, 27, 28, 29, 30, 31]. A detailed summary of the search process and results is presented in Figure 1.

### 3.2 Characteristics of the Included Studies

These studies, except for one prospective study, were all retrospective case–control studies, published between 2016 and 2023. All the studies compared outcomes between miscarriage and nonmiscarriage cases. The age range of patients included in these studies was between 18 and 42 years. The BMI of the patients did not exceed 30 kg/m^2^. Thirteen studies were from Turkey, with one each from China, Iran, and Korea. The gestational age in the studies reviewed was determined using the last menstrual period and subsequently verified through crown‐rump length measurements obtained via abdominal or transvaginal ultrasound. Participants with inadequate data, uterine structural anomalies, acute or chronic infectious diseases, cancer, comorbidities under medical treatment, and those who smoked during pregnancy were excluded from the study. Additionally, two of the studies excluded cases with chromosomal abnormalities [15, 28]. Detailed information about the included studies can be found in Table 1.

**Table 1:** Baseline characteristics of include studies and methodological assessment.

Authors	Study period	Country	Study design	Types of miscarriage	Patients (*n*)		Timing (week)	Age (year)	BMI (Kg/m^2^)	Quality score
					miscarriage	Control				
Yavuz et al.	2018.1–2021.11	Turkey	Retrospective	Miscarriage	50	50	6–13	18–42	/	9
Oglak et al.	2019.9–2019.12	Turkey	Retrospective	Early pregnancy loss	137	148	≤ 13	18–35	23.12 ± 3.66/23.78 ± 3.82	8
Akin et al.	2012.8–2014.8	Turkey	Retrospective	Miscarriage	78	91	First trimester	30.3 ± 6.8/28.1 ± 5.4	/	7
Yakistiran et al.	2019.9–2020.1	Turkey	Retrospective	Miscarriage	193	164	6–10	27.8 ± 5.6/30.6 ± 6.8	/	8
Yakistiran et al.	2019.9–2020.1	Turkey	Retrospective	Elective miscarriage	32	164	6–10	31.1 ± 6.7/30.6 ± 6.8	/	8
Ata et al.	2018.1–2019.5	Turkey	Retrospective	Early pregnancy loss	100	100	7–14	27.7 ± 4.7/27.1 ± 5.2	/	8
Sert et al.	2018.1–2021.12	Turkey	Retrospective	Missed miscarriage	142	142	7–12	28.7 ± 6.9/27.1 ± 5.2	/	7
Gorkem et al.	2018.9–2019.8	Turkey	Prospective	Spontaneous miscarriage	30	30	7–8	20–35	20–30	7
Hivehchi et al.	2021.3–2022.3	Iran	Retrospective	Missed miscarriage	120	120	6–13	18–42	< 25	7
Bas et al.	2012.1–2017.1	Turkey	Rretrospective	Miscarriage	173	245	< 14	31.88 ± 6.43/30.15 ± 5.62	< 25	8
Bas et al.	2012.1–2017.1	Turkey	Retrospective	Miscarriage	152	245	14–20	30.87 ± 6.19/30.15 ± 5.62	< 25	8
Biyik et al.	2015.1–2018.12	Turkey	Retrospective	Missed miscarriage	40	40	6–14	17–42	< 25	8
Liu et al.	2018.1–2020.12	China	Retrospective	Missed miscarriage	200	200	6–12	18–35	22.14 ± 2.69/21.96 ± 2.15	8
Uysal et al.	2014.4–2014.12	Turkey	Retrospective	Missed miscarriage	90	143	6–16	27.2 ± 6.7/26.7 ± 5.7	24 ± 3/24 ± 4	8
Kim et al.	2011.1–2018.12	Korea	Retrospective	Missed miscarriage	72	104	< 14	33.08 ± 4.23/32.53 ± 6.08	/	7
Soysal et al.	2019.1–2020.12	Turkey	Retrospective	Miscarriage	43	107	6–19	/	/	8

### 3.3 Quality Assessment of Included Studies

The NOS was utilized to evaluate the quality of the included studies [32]. Studies scoring seven to nine points were regarded as high quality [19]. Each study was eligible to earn a maximum of one star for each item within the Selection and Exposure categories, and up to two stars for Comparability. The quality scores for the included studies are presented in Table 1.

### 3.4 Disparities in PLR Between the Miscarriage and Nonmiscarriage Groups

**Figure 2:**
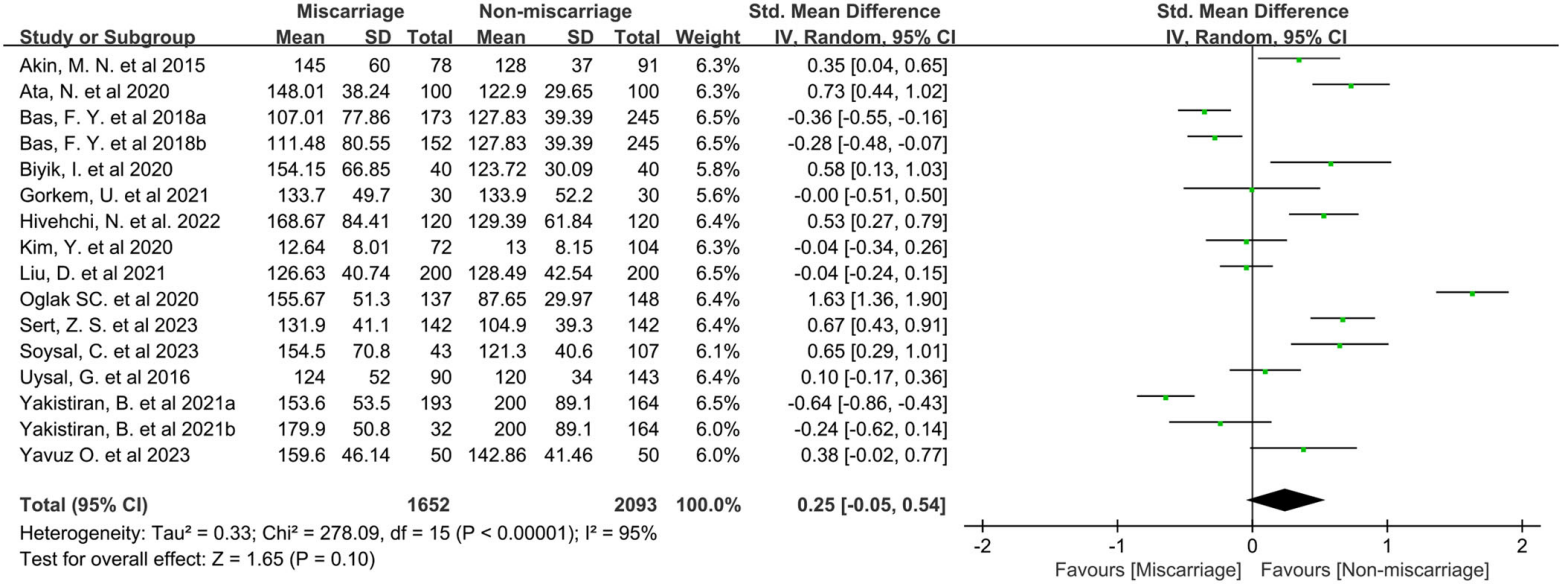
Meta‐analysis forest plot for PLR level.

**Figure 3:**
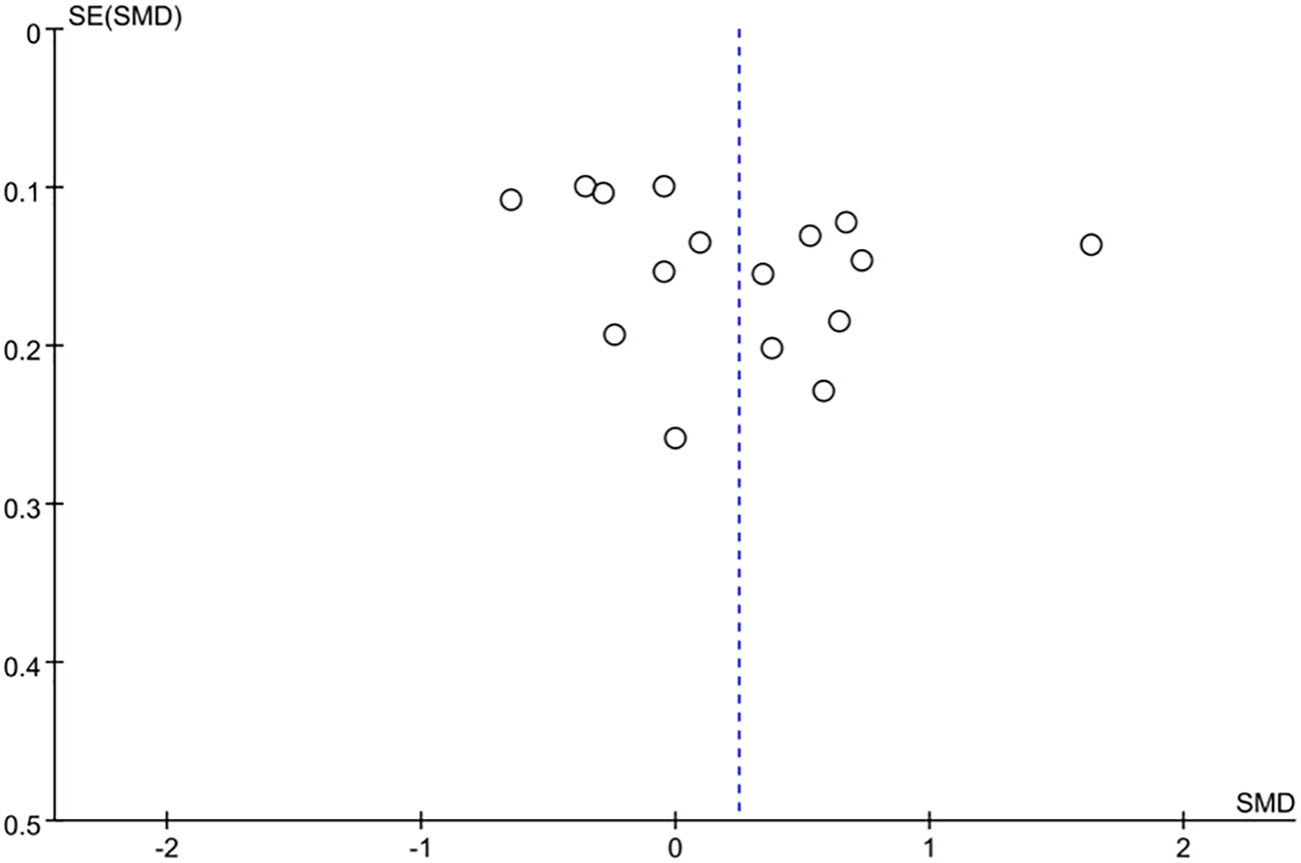
Meta‐analysis funnel plots for PLR level.

**Figure 4:**
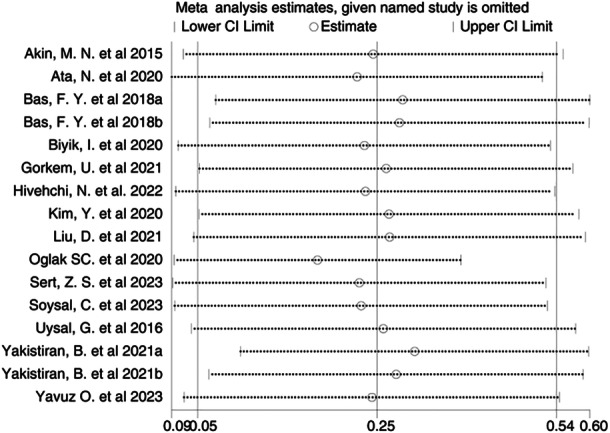
Sensitivity analysis for PLR level.

To examine the potential of PLR as a predictor of miscarriage risk, a meta‐analysis was conducted including 16 studies from 14 articles. The analysis compared PLR values between miscarriage cases and controls, encompassing 3745 participants (1652 miscarriage cases and 2093 controls). Results showed comparable PLR levels between the miscarriage and the nonmiscarriage groups (SMD = 0.25; 95% confidence interval (CI): −0.05 to 0.54). Significant heterogeneity was observed (*I* ^2^ = 95%, *p *< 0.00001) (Figure 2). The funnel plot (Figure 3) and Egger's test (*p* = 0.167) indicated no significant publication bias. However, sensitivity analysis indicated instability in the results; the exclusion of the study by Yakistiran et al. [17] resulted in a shift from nonsignificant to significant findings (SMD = 0.31; 95% CI: 0.24–0.59) (Figure 4).

**Figure 5:**
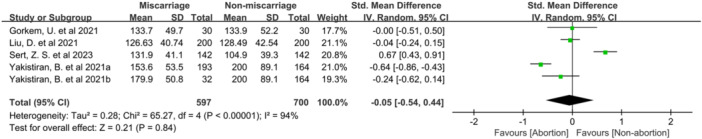
Meta‐analysis forest plot for PLR level of early miscarriage.

A subgroup analysis of early miscarriage (< 12 weeks gestation) included five studies from four articles [17, 23, 24, 28]. This analysis showed comparable PLR levels between groups (SMD = −0.05; 95% CI: −0.54 to 0.44), with substantial heterogeneity (*I* ^2^ = 94%, *p *< 0.00001) (Figure 5).

### 3.5 The Relationship Between PLR Levels and the Risk of Miscarriage

**Figure 6:**
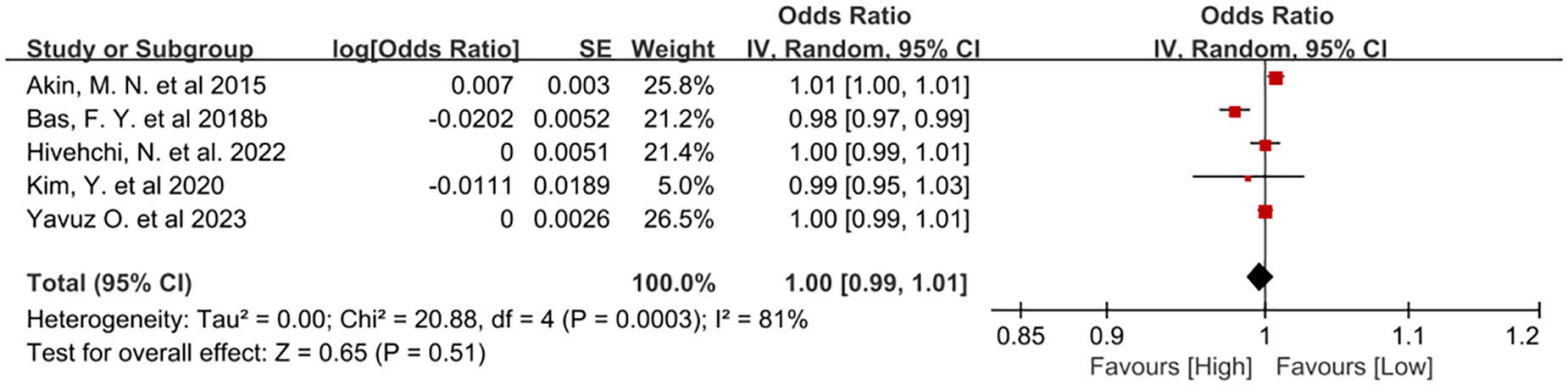
Meta‐analysis forest plot for risk of miscarriage.

**Figure 7:**
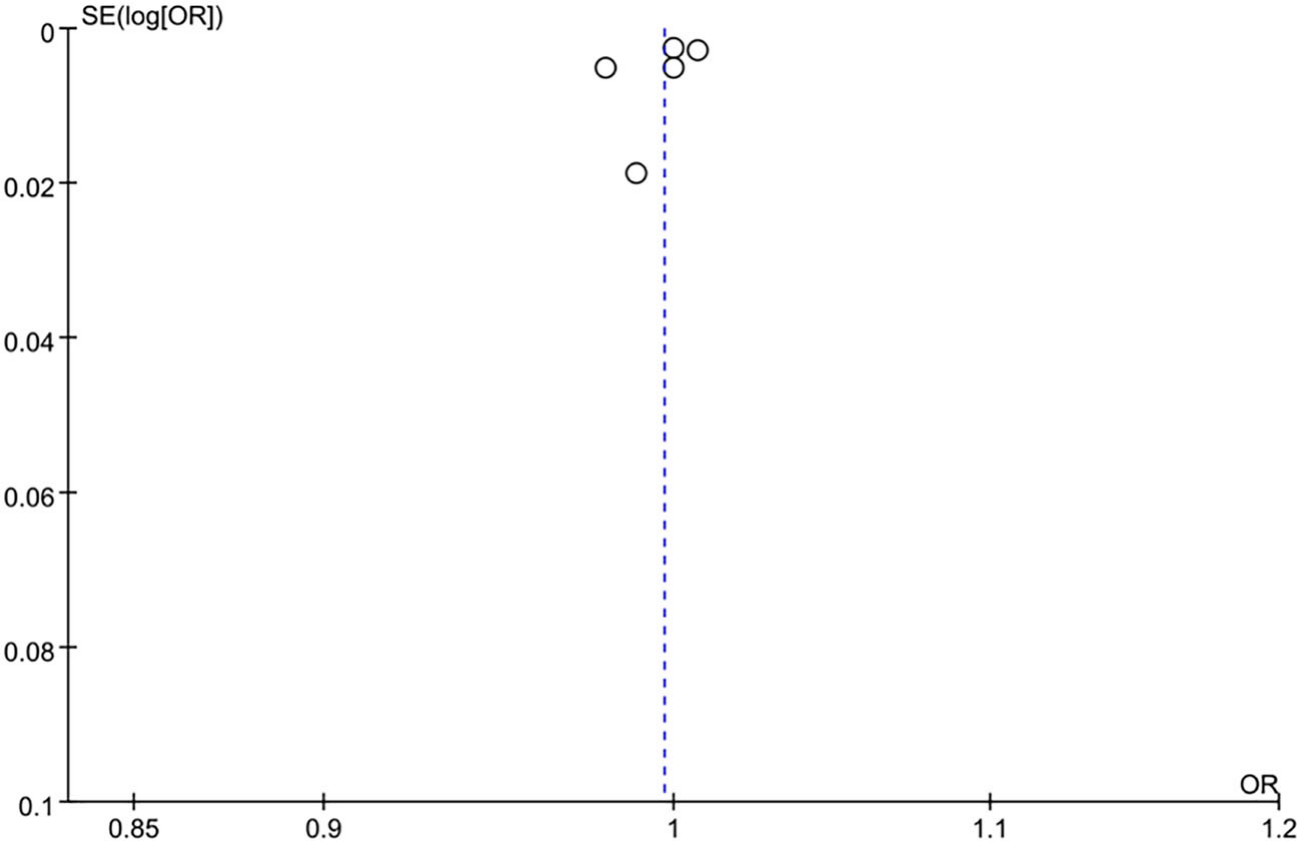
Meta‐analysis funnel plots for risk of miscarriage.

**Figure 8:**
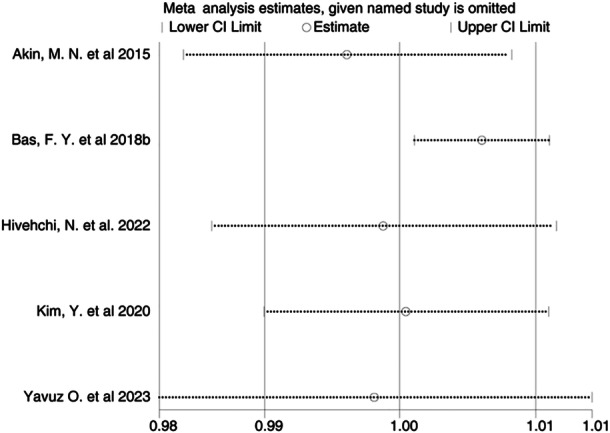
Sensitivity analysis for risk of miscarriage.

Five studies employed logistic regression to examine whether elevated PLR was a risk factor for miscarriage [15, 21, 25, 26]. Analysis was conducted using a random‐effects model due to significant heterogeneity (*I* ^*2*^ = 81%, *p *= 0.0003). The pooled analysis showed no statistically significant combined effect (OR = 1.00; 95% CI = 0.99–1.01) (Figure 6). Publication bias was not evident in the funnel plot (Figure 7) or Egger's test (*p *= 0.431). Sensitivity analysis confirmed the stability of these results (Figure 8).

### 3.6 Subgroup Analysis

Subgroup analyses were performed to evaluate PLR levels and miscarriage risk across different countries, sample sizes, and miscarriage types. Results showed significantly higher PLR levels in miscarriage cases compared to controls in two subgroups: the missed miscarriage group (SMD = 0.29; 95% CI: 0.01–0.56) and studies with sample sizes of less than 200 (SMD = 0.31; 95% CI: 0.05–0.56). No significant differences were observed in other subgroups. The subgroup analysis of PLR levels in relation to the risk of miscarriage indicated that none of the subgroups were statistically significant (Table 2).

**Table 2:** Subgroup analysis.

Subgroup	PLR level				Risk of miscarriage			
	Study	MD [95% CI]	*p* value	*I* ^2^	Study	OR [95% CI]	*p* value	*I* ^2^
Total	16	0.25 [−0.05, 0.54]	0.1	95%	5	1.00 [0.99, 1.01]	0.51	81%
Types of miscarriage								
Missed miscarriage	6	0.29 [0.01, 0.56]	0.04	84%	2	1.00 [0.99, 1.01]	0.88	/
Miscarriage	9	0.27 [−0.23, 0.77]	0.2	97%	3	1.00 [0.98, 1.01]	0.51	90%
Elective miscarriage	1	−0.24 [−0.62, 0.14]	0.22	/	/	/	/	/
Sample size								
> 200	8	0.20 [−0.27, 0.66]	0.41	97%	2	0.99 [0.97, 1.01]	0.32	87%
≤ 200	8	0.31 [0.05, 0.56]	0.02	75%	3	1.00 [1.00, 1.01]	0.35	45%
Country								
Turkey	13	0.27 [−0.09, 0.64]	0.15	95%	3	1.00 [0.98, 1.01]	0.57	90%
Iran	1	0.53 [0.27, 0.79]	< 0.0001	/	1	1.00 [0.99, 1.01]	1	/
China	1	−0.04 [−0.24, 0.15]	0.66	/	/	/	/	/
Korea	1	−0.04 [−0.34, 0.26]	0.77	/	1	0.99 [0.95, 1.03]	0.56	/

## 4 Discussion

Miscarriage is a prevalent pregnancy complication with significant physical and psychological impacts [33]. Human pregnancy involves the implantation of a semiallogeneic fetus into the endometrium, requiring precise physiological regulation of immune responses to prevent rejection [33, 34]. Systemic inflammation is a crucial factor throughout pregnancy, with elevated levels observed during this period [35]. Inflammation, in conjunction with the immune system, plays a pivotal part in normal pregnancy, from implantation to parturition. This interaction is a current focal point of research among various other mechanisms. The reduction in lymphocyte count occurs as a physiological immune response in numerous systemic inflammatory diseases and malignancies [36]. Platelets, while essential for hemostasis, so contribute significantly to inflammation by binding to the endothelium and activating lymphocytes. Studies have demonstrated that PLR serves as a systemic inflammatory marker in various conditions, including prothrombotic, rheumatologic, atherosclerotic, metabolic, and oncologic diseases [32, 37, 38]. As an indicator of systemic inflammation and stress [8, 39], PLR offers a more accessible and cost‐effective alternative to other serum markers associated with miscarriage, such as periodin, Th‐1, TNF‐α [20], and Th‐17 [40].

Currently, there are no standardized biomarkers for predicting miscarriage, and the clinical significance of PLR in this context remains unclear. This study presents the first systematic review and meta‐analysis examining PLR's predictive value for miscarriage. Our findings suggest that PLR does not have significant predictive value for miscarriage risk. However, sensitivity analysis revealed that the overall results were unstable. Exclusion of the study by Yakıstıran [17] led to statistically different results. These discrepancies may be attributed to factors such as miscarriage type and sample sizes. In the study of Yakıstıran and colleagues from 193 cases of spontaneous miscarriage and 164 healthy pregnancies, it was observed that PLR levels were lower in the spontaneous miscarriage group.

The subgroup analysis by sample size revealed that the prognostic value of PLR in predicting miscarriage was significant when the sample size was 200 or less. However, with larger sample sizes, the reliability of the findings improveed, rendering the result clinically insignificant. In the subgroup analysis by countries, a study conducted in Iran with a sample size of 240 pregnant women (120 cases of missed miscarriage and 120 cases of normal pregnancies) reported significantly higher PLR values in women with missed miscarriage compared to those with normal pregnancies [25]. It is noteworthy that the miscarriage type investigated in this study was specifically missed miscarriage.

Missed miscarriage is a distinct type of miscarriage marked by immune system dysfunction and imbalance, in which the embryo or fetus dies but remains in the uterus without natural expulsion [41]. It can occur without obvious symptoms and affects about 8%–20% of clinically diagnosed pregnancies [42]. Although the pathogenesis of missed miscarriage has been extensively studied in obstetrics, the correlation between PLR values and missed miscarriage remains incompletely elucidated [43]. Among the 16 studies reviewed, 5 explicitly focused on women with missed miscarriage as their study cohort [23, 27, 28, 29, 30]. Additionally, in one study [25], the term “miscarriage” was used to represent missed miscarriage, defined as intrauterine death without expulsion.

Approximately 80% of miscarriages occur within the initial 12 weeks of pregnancy, with the risk decreasing significantly after this period [4]. To examine this further, we conducted a more detailed analysis focusing on studies involving a gestational age of 12 weeks or less [17, 23, 24, 28]. The findings revealed that PLR values do not have predictive significance for early miscarriage.

In the study by Sert and Bulbul [23], the research population was categorized into two groups: missed miscarriage and healthy pregnancies. The results showed that the average PLR value was significantly higher in the missed miscarriage group compared to the healthy pregnancy group (131.9 ± 41.1 vs*.* 104.9 ± 39.3, *p* < 0.001), suggesting a potential relation between PLR levels and the incidence of missed miscarriage. Logistic regression analysis identified PLR as an independent predictor of missed miscarriage (OR = 2.15, 95% CI: 1.05–2.78, *p* = 0.019). Furthermore, the diagnostic accuracy of PLR was assessed using Receiver Operating Characteristic (ROC) curve analysis, which yielded an area under the curve (AUC) of 0.711, suggesting a moderate predictive ability of PLR for missed miscarriage. Consistent with these results, our study also observed higher PLR values in individuals with missed miscarriage.

Another study focusing on individuals with missed miscarriage between gestational weeks 6–14 revealed elevated PLR values in the missed miscarriage group. Nevertheless, the difference was not statistically significant compared to the control group (*p* = 0.3), implying that PLR may not be a reliable indicator for predicting missed miscarriage [15]. Despite this, subgroup analysis within our research revealed notably higher PLR values in the missed miscarriage group in contrast to healthy pregnant individuals. As a meta‐analysis, our findings carry greater statistical weight than individual studies, supporting the conclusion that PLR can serve as a marker for predicting missed miscarriage.

In the meta‐anlysis of Sedigheh Hantoushzadeh and colleagues, which included 14 studies, no significant difference in PLR was found between the pregnancy loss group and the control group. However, their analysis did not separately examine early pregnancy losses, even though most miscarriages occur during early pregnancy. Moreover, recurrent miscarriage, missed miscarriage, and threatened miscarriage may have different pathological and physiological mechanisms. In our study, subgroup analyses of different types of miscarriage were conducted, and the results demonstrated that PLR has predictive value specifically for missed miscarriage. In addition, our analysis included more studies, providing stronger evidence for this conclusion [14].

To summarize, missed miscarriage is associated with immune system dysfunction and imbalance, and elevated PLR levels may serve as a useful and affordable marker for missed miscarriage prediction. However, PLR is unable to forecast the risk of other types of miscarriage. PLR is an indicator of inflammation and immunity that may be more predictive of cancers. Furthermore, this study recorded static PLR values at a specific point during pregnancy, without analyzing temporal variations over its course. The main conclusions of our article are applicable in the clinical setting for identifying high‐risk patients who are prone to missed miscarriage. Early identification could enable proactive intervention, potentially lowering the miscarriage rate. Besides, the findings could assist in developing predictive models to identify high‐risk populations during pregnancy.

The primary strength of this study lies in its novelty as the first systematic review and meta‐analysis to evaluate the predictive value of PLR for miscarriage. Multiple lines of evidence indicate that PLR lacks predictive potential for miscarriage overall, highlighting the need for caution to avoid its inappropriate application in clinical practice. However, this study is not without limitations. First, most of the included studies were retrospective, with only one prospective study, which increased the risk of confounding and bias. Second, the predominantly Asian study population limited the generalizability of findings due to potential selection bias and uncertain applicability to other populations. Third, certain indicators showed significant heterogeneity and instability.

Given the diverse etiologies of miscarriage and limited chromosomal analyses of aborted tissues, it is crucial for future large‐scale, case–control studies to focus specifically on missed miscarriage, excluding embryos with chromosomal abnormalities. Prospective, multicenter studies with larger and more diverse populations are essential to fully determine the predictive value of PLR for miscarriage, as well as to establish its optimal use within clinical settings. Such research could help clinicians identify high‐risk pregnancies earlier and design effective interventions to reduce miscarriage rates.

## 5 Conclusion

This meta‐analysis demonstrates that PLR is not a significant predictor of miscarriage overall. However, in cases of missed miscarriage, elevated PLR levels may hold predictive value.

## Author Contributions

**Xiaoyi Wang:** conceptualization, data curation, formal analysis, investigation, methodology, software, writing–original draft, writing–review and editing. **Yanfang Zhao:** data curation, investigation, writing–original draft. **Yun Liu:** funding acquisition, project administration, supervision, writing–review and editing. **Yangyang Fan:** project administration, supervision, writing–review and editing.

## Ethics Statement

This study was a review and meta‐analysis of existing, published literature not requiring ethics committee approval.

## Consent

The authors have nothing to report.

## Conflicts of Interest

The authors declare no conflicts of interest.

## Supporting information

Supporting information.

## Data Availability

The data sets used and analyzed during the current study are available from the corresponding author on reasonable request.
