# Supplementary material for: The Predictive Significance of Platelet‐to‐Lymphocyte Ratio for Miscarriage: A Systematic Review and Meta‐Analysis
Source: Immun Inflamm Dis. 2025 Jan 7;13(1):e70119. doi: 10.1002/iid3.70119 (PMC11705396; doi:10.1002/iid3.70119)
Supplement: Supplementary file 1 — Supporting information. [file IID3-13-e70119-s001.docx]

Pubmed -21

(((Blood Platelets[MeSH Terms])OR("Blood Platelet "[Title/Abstract]OR"Platelet,Blood "[Title/Abstract]OR"Platelets,Blood"[Title/Abstract]OR "Thrombocytes "[Title/Abstract]OR"Thrombocyte "[Title/Abstract]OR "Platelets "[Title/Abstract]OR"Platelet "[Title/Abstract]))AND((Lymphocytes[MeSH Terms])OR((Lymphocyte)OR(Lymphoid Cells)OR(Cell,Lymphoid)OR(Cells,Lymphoid)OR(Lymphoid Cell ))))AND(ratio))AND((Miscarriage)OR(Abortion ))

Embase-2


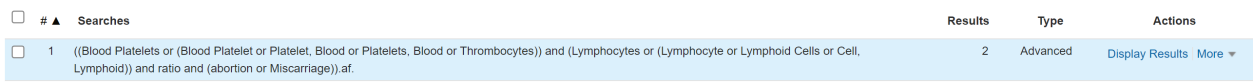


Cochrane -0


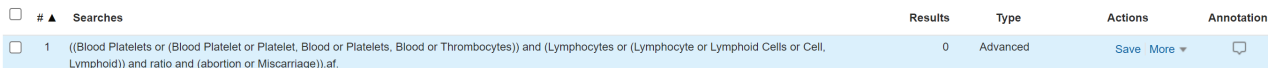


Wos-31

((((Blood Platelets) OR ((((Blood Platelet) OR (Platelet, Blood)) OR (Platelets, Blood)) OR (Thrombocytes))) AND ((Lymphocytes) OR (((Lymphocyte) OR (Lymphoid Cells)) OR (Cell, Lymphoid)))) AND (ratio)) AND ((abortion) OR (Miscarriage)) (Topic)
